# Functional Capacity and Motor Performance of Upper Limbs in Individuals with Cerebellar Disorders: A Pilot Study

**DOI:** 10.1155/2017/8980103

**Published:** 2017-08-08

**Authors:** Vivian Farahte Giangiardi, Sandra Maria Sbeghen F. de Freitas, Flávia P. de Paiva Silva, Renata Morales Banjai, Sandra Regina Alouche

**Affiliations:** Master's and Doctoral Program in Physical Therapy, Universidade Cidade de São Paulo, São Paulo SP, Brazil

## Abstract

In simple daily activities carried out by the upper limbs, the cerebellum is responsible for the adaptations required for the accurate movement based on previous experiences and external references. This paper aims to characterize the performance of the upper limbs after a cerebellar disease. We evaluated the digital and handgrip strength, dexterity, and function of the upper limbs. The motor performance of the upper limbs was assessed through the use of a digitizing tablet by performing aiming movements with the upper limb most affected by cerebellar disease and the paired limb of the healthy group. The results showed differences between groups: the cerebellar group had higher latency to movement onset, was slower, and presented less smooth trajectories and higher initial direction errors. Moreover, the movement direction influenced the peak velocity and the smoothness for both groups (contralateral directions were slower and less smooth). We concluded that cerebellar disorder leads to movement planning impairment compromising the formulation of an internal model. Alterations on movement execution seem to be a consequence from disruptions in the anticipatory model, leading to more adaptations. These findings are compatible with the roles of the cerebellum on the control of voluntary movement.

## 1. Introduction

The role of cerebellum in planning and execution of movements is well known [1]. Motor planning will occur through connections with the cerebral cortex associated to sensory information and cognitive commands [2, 3]. This association will constitute the basis for the formulation of an anticipated model, which links the motor commands with the prediction of the sensory consequences of movement (forward model) [3, 4]. Besides that, the cerebellum establishes parameters during the execution of voluntary movements and makes adjustments to unexpected conditions [5]. In general, these adjustments will be made through the use of external references based on previous experiences [4]. In this way, the cerebellum allies what was predicted and what has been done, adapting movements according to the environment [6] and assuring their accuracy and smoothness [7].

Cerebellar damage or damage of its connections with other structures leads to sensorimotor deficits [8]. The movements of the upper limbs performed by individuals with cerebellar disorders are characterized by greater latency and slower execution [1], which reflect the attempts of the damaged cerebellum to compare the planned forward model with the sensory information received during the movement execution [9]. Such inability may result in deficits in the modulation of the forces required for reaching and manipulation of objects with accuracy [10]. Thus, making corrections and errors are more often. Studies that analyze the adaptations on aiming movements in this population in response to external perturbations demonstrate that cerebellar dysfunction leads to impaired movement, characterized by slower and less precise execution [3, 4, 11, 12].

The direct consequences of cerebellar damage in the control of aiming movements, during movement planning (forward model) or execution (adaptations), have been explored in isolation [4, 13]. It has been clear, for many years, what the cerebellum's role is in the execution of movements. The cerebellum is known as the lead structure that makes corrections (adaptations) while the movement is executed [14]. Although considering the findings with regard to the cerebellum's roles in the anticipation of movements, and in this way, planning the movement, it is still unclear which factor, anticipation or adaptation, represents the main role of this structure on the control of voluntary limb movements. Is the cerebellum primarily responsible to plan smooth and coordinated movement, directing motor neurons accordingly, or to assure that the motor system uses the available sensory information to execute movements, including smooth and coordinated movements? The investigation of factors involved in the cerebellum control of arm movements is necessary. This knowledge will help to demonstrate the consequences of cerebellar damage on the upper limb control and the establishment of a comprehensive assessment of the clinical profile, functional capacity, and motor performance of the upper limbs in this population. Therefore, this pilot study aimed to characterize the functional capacity and motor performance of the upper limbs in aiming movements in individuals with cerebellar disorders.

## 2. Materials and Methods

A cross-sectional, pilot study was conducted in a movement analysis laboratory with partial soundproofing. All procedures were approved by the Ethics Committee for Institutional Research and followed Helsinki Declaration principles.

Twelve participants were enrolled in the study. Individuals diagnosed with cerebellar damage of vascular (ischemia or hemorrhage) or degenerative nature for more than 6 months were included in the cerebellar group (CbG, *n* = 6). Participants in the healthy group (HG, *n* = 6) were matched according to age and sex with those of the cerebellar group. All participants were adults over 18 years old and right-handed, established through the *Edinburgh Inventory* [15]. Individuals with other neurological diseases, musculoskeletal disorder of the upper limb or pain, and clinical instability and clinical signs of the peripheral nervous system by *diabetes mellitus* were excluded from this study. Sociodemographic, clinical, and physical-functional data of the participants are presented in Table 1.

### 2.1. Procedures

Participants were informed about the study procedures and signed the informed consent. Their sociodemographic data and clinical-functional tests were collected (Table 1). The motor and sensorial (superficial and profound) functions of the upper extremity as well as the motor coordination (evaluation used to identify the most affected upper limb by the cerebellar disorder) [16] were evaluated.

The handgrip strength was obtained (kgf) through a SH5001 manual dynamometer model (Saehan®), and the pinch strength by Pinch Gauge (*Jamar*®) dynamometer through the junction of the thumb and index finger pulps [17]. Three consecutive trials were performed in both upper limbs, and the average across trials was considered for analysis. *Purdue Pegboard Test* was used to assess dexterity [18] in individuals with cerebellar disorders. The test was performed with the most affected upper limb. Participants should fit the maximum number of pins to their holes, over a period of 30 seconds. The scoring was based on the number of pins that the participant fitted in the proposed time [19]. The results were compared with normative results considering the age of the studied sample [18].

**Figure 1:**
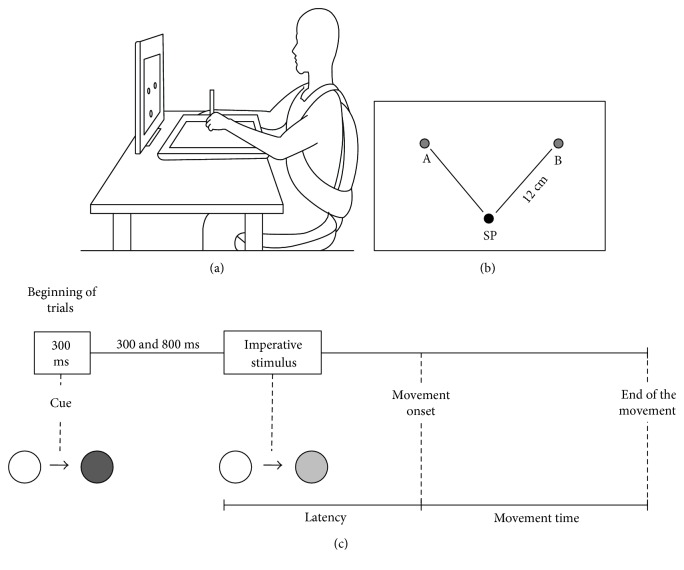
Representation of participant's position (a). Position of the targets: SP—starting point, target A— positioned 45° to the left of the SP, and target B—positioned 45° to the right of the SP (b). Representation of task events in a trial (c) during the aiming movements. Cue—change of the target color, from white to red (direction of movement). Imperative stimulus—change of the target color, from white to green (movement could start).

For the evaluation of the upper limb performance in aiming movements, participants of the cerebellar group used the most affected upper limb by the cerebellar disorder. The side used by the healthy group was matched to the limb used by the cerebellar group. Participants were asked to perform movements directed to targets using a stylus from a predetermined starting point over the sensitive surface of a tablet (WACOM Intuos® 2.12 × 12 inches). Three targets were presented on the monitor screen (Samsung®, 15 inches) which was positioned at eye level (Figure 1(a)). The lower target, which was centered on the screen and aligned with the participant's body, was considered the starting point (SP). The other two targets were positioned at 45° to the left and right of SP, at a distance of 12 cm. All targets were 1 cm in diameter. Thus, the movements were performed for targets positioned on the same side (ipsilateral) or opposite side (contralateral) of the moving upper limb (Figure 1(b)). All devices were connected to a laptop (HP AMD Turion® 64) that controlled the tasks through the Software LabView 9.0®, which was also used for data analysis.

After the positioning of the stylus in the SP, a stimulus that signaled the direction of the movement (change of target color from white to red) that lasts 300 ms was presented. After an interval ranging between 300 and 800 ms, the target changed from white to green (imperative stimulus) indicating that the participant should start the movement (Figure 1(c)). The instruction given to all participants before the task was to “reach the center of the target as fast and accurate as possible.” Four blocks with 20 valid trials for each target were executed randomly, and the trials were restored at the end of the block in case of errors.

### 2.2. Data Analysis

Sociodemographic, clinical, and functional data of the studied groups were analyzed by using descriptive statistics for quantitative variables and through frequency analysis for qualitative variables. The physical and functional data obtained for each group were compared by using Mann–Whitney *U* test when the variables were continuous.

The trajectory of the stylus tip over the digitizing tablet (*X* and *Y* coordinates) during aiming movements was collected at a frequency of 300 Hz. A low-pass Butterworth filter at 10 Hz was used. The resulting linear velocity of the trajectory was calculated and its peak velocity found, in cm/s. The beginning and end of each trial were considered the moment 5% of peak velocity was reached. Temporal and spatial variables were evaluated. Reaction time was considered the interval between the imperative stimulus and the beginning of the movement, measured in milliseconds. Movement time was the time between the beginning and the end of the movement, measured in milliseconds. The smoothness was the number of times that the acceleration curve changed direction, measured as movement unities (mu). The initial direction error was the trajectory deviation in degrees from the straight line between SP and the target, in the first acceleration peak; and the resulting variable error was measured as the position difference between the end point of the movement of each trial and the final position error. The average of all trials for each participant was calculated and used for analysis.

The averaged values by the participants in each condition were subjected to nonparametric analysis due to the restricted sample of this pilot study. The exact test was used for all analysis to be more accurate. Differences between groups (healthy – HG × cerebellar − CbG) and directions (ipsilateral and contralateral movements) were verified. The significance value was set at 5%. All analyses were performed using IBM® SPSS Statistics package, version 19.0.

## 3. Results

**Figure 2:**
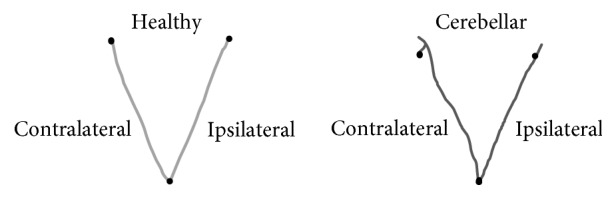
Typical trajectory made by the right upper limb of one healthy individual and one individual of the cerebellar group.

A typical trajectory made by one individual of the healthy group and cerebellar group is presented in Figure 2.

**Figure 3:**
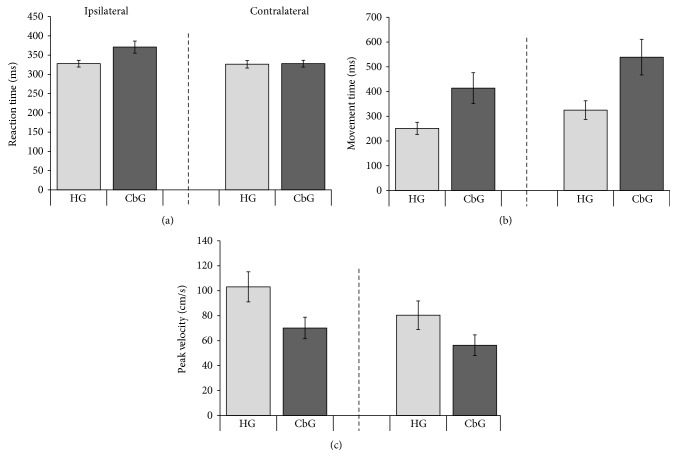
Reaction time (a) in milliseconds (ms), movement time (b) in milliseconds (ms), and peak velocity (c) in centimeters per second (cm/s) of the cerebellar (CbG) and healthy (HG) groups in aiming movements. Ipsilateral—the same side of the moving arm; contralateral—opposite side of the moving arm (average and standard error).

The results of temporal variables obtained in aiming movements will be described by the median for each group, which is more appropriate for nonparametric statistics. The CbG (362 ms) had longer reaction time than the HG (320 ms) in the ipsilateral (*U* = 4.00, *p* = 0.026, *r* = −0.64) direction but not in the contralateral (*U* = 8.00, *p* = 0.13, *r* = −0.46) direction (HG = 318 ms; CbG = 362 ms; Figure 3(a). No differences in reaction time between directions were found (*T* = 21; *p* = 0.18). The HG had shorter movement time (Figure 3(b)) than the CbG for both ipsilateral (HG = 256 ms; CbG = 410 ms; *U* = 5.00, *p* = 0.04, *r* = −0.61) and contralateral (HG = 342 ms; CbG = 539 ms; *U* = 6.00, *p* = 0.05, *r* = −0.55) directions. Contralateral movements were longer (393 ms) than the ipsilateral movements (294 ms) for the two groups (*T* = 0; *p* < 0.0001).

The peak velocity was similar between groups for both ipsilateral (HG = 96 cm/s; CbG = 69 cm/s; *U* = 7.00, *p* = 0.93, *r* = −0.49) and contralateral directions (HG = 74 cm/s; CbG = 52 cm/s, *r* = −0.49) (Figure 3(c)), but ipsilateral movement presented a higher peak velocity (85 cm/s) than the contralateral movement (60 cm/s) for both groups (*T* = 0; *p* < 0.0001).

**Figure 4:**
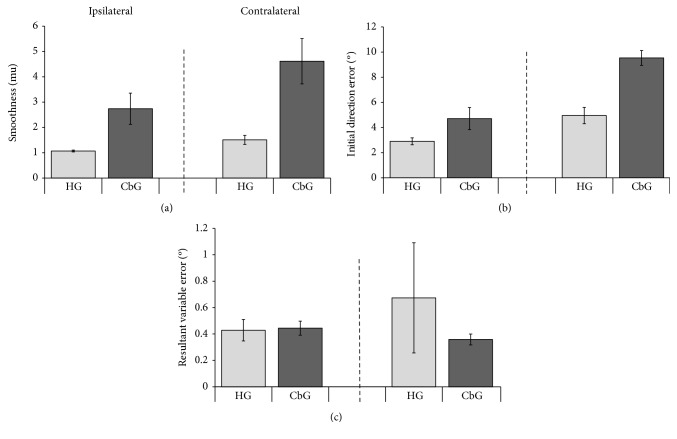
Smoothness (a), initial direction error (b), and resultant variable error (c) in centimeters (cm) of the cerebellar and healthy groups in aiming movements. Ipsilateral—the same side of the moving arm; contralateral—contralateral side of the moving arm; HG (healthy group) and CbG (cerebellar group) (average and standard error).

Regarding the spatial variables, there was a difference between groups in the movement smoothness for both ipsilateral (*U* = 1.00, *p* = 0.04, *r* = −0.78) and contralateral movements (*U* = 3.00, *p* = 0.015, *r* = −0.69; Figure 4(a) and in the initial direction error for contralateral movements (ipsilateral: *U* = 10.00*p* = 0.20, *r* = −0.37; contralateral: *U* = 0.00, *p* = 0.002, *r* = −0.83; Figure 4(b)). The CbG was less smooth and made more errors than the HG. In addition, the contralateral movement was less smooth (*T* = 0; *p* = 0.001) and showed higher errors (*T* = 1.00; *p* = 0.001) than the ipsilateral movements.

There were no differences between groups (Figure 4(c)) in the resulting variable error for both directions (ipsilateral: *U* = 17.00, *p* = 0.94, *r* = −0.16; contralateral: *U* = 13.00, *p* = 0.49, *r* = −0.23) and in the final position error (ipsilateral: *U* = 11.00, *p* = 0.31, *r* = −0.32; contralateral: *U* = 17.00, *p* = 0.94, *r* = −0.05). No differences were observed between ipsilateral and contralateral directions for both variables (*T* = 17.00; *p* = 0.02) and in the final position (*T* = 37.00; *p* = 0.91) error.

## 4. Discussion

This study aimed to characterize the functional capacity and motor performance of the upper limbs in patients with cerebellar disorder. Overall, the study demonstrated that individuals with cerebellar damage showed lower dexterity and changes in the planning and execution of aiming movements. These findings highlight the role of the cerebellum in the formulation of an anticipatory model that predicts the sensory consequences of movements and the adjustments required for the accurate movement.

Cerebellar damage led to a worse performance in the assessed manual in comparison to healthy individuals. In the age group of the studied population (35 or older), a range of 12 to 15 hits in both upper limbs are described [19]. Individuals with cerebellar disease had an average of 6–9 hits, which is 56.6% less hit than those of healthy individuals. These data can be related to the change in the prediction of the strength required for handling objects. Nowak et al. [10] described that the coordination of grip in handling objects is the most affected aspect in fine motor control of patients with cerebellar disease. These authors suggested that the modulation loss is directly related to changes in the formulation of the forward model, which links the motor planning with the prediction of the sensory consequences of the movement [3, 4, 10].

The consequences of the impairment caused by the cerebellar damage were observed, in the present study, considering planning and execution phases. During the execution, movements were less smooth and took longer to be completed, which means that individuals made more corrections to complete movements in an accurate and precise way. Aiming movement starts with a submovement directed to the center of the target. The higher speed and muscular strength employed for the beginning of the movement caused the initial direction error associated with this submovement [20]. When this submovement exceeds or falls below the target range, a correction is necessary to a second submovement, which may not be sufficient to reach the target. As the sensory information provided to the participant does not match the performed movement, participants needed to modify their movements to produce the appropriate result. [21]. Bastian [22] postulated that individuals with cerebellar disorders use more submovements to reach the target, with slower movements and higher frequency of correction, since the sensorial information is integrated in a deficient manner [22]. However, when considering all execution variables, differences regarding the resulting variable error and the final position error were not found, which may suggest that these individuals were able to accomplish the final goal of the task and reach the target.

The notion of an impaired planning phase of the movement is confirmed by other results of the aiming movement performance. Individuals with cerebellar disorders had longer movement onset and higher initial direction error (especially for ipsilateral movements) than healthy individuals. Miall and King [23] showed that cerebellar activation occurs prior to the start of the movement (between 120 and 140 ms). It is suggested that such activation is related to the interpretation of somatosensory information of the position of limbs and characteristics of the task [23]. A communication error caused by a cerebellar damage will make an impairment in this interpretation and generate a deficient motor planning [23]. It is postulated that corrections during movement execution will take place after a period of 200 ms; before this time interval, the movement is based on motor planning [20]. The attempt to compensate the initial failures of planning requires more sensory feedbacks and corrections evidenced, in this study, by the increased duration of the movement (200 to 600 ms). These corrections will increase the duration of the movement, promoting more need for adaptations [24].

The deceleration phase of the movement, which happens after the peak velocity till the end of the movement, is regulated by the constant feedback of somatosensorial [20] and visual [6] sources. The cerebellum is the structure responsible for interpreting the somatosensory feedback, by promoting the adjustments from movements previously performed [3]. Cerebellar damage and its communications cause malfunctions in the interpretation of this feedback and in the formulation of anticipatory model for voluntary movement [11]. If the required adjustments and corrections in the continuous regulation of the movement [25] are not performed efficiently [1], the duration of the movement will increase and the number of corrections required will be higher [11, 22, 25], as seen in this study. Therefore, the coordination of the limb movement depends on the cerebellar integrity. In this way, the upper limb coordination will occur as a result of the previously formulated internal model associated to the information arising from the internal feedback and sensory consequences expected after the motion planning [22].

Considering the results, it can be postulated that impairments in aiming movements were more substantial for the planning, and by this impairment in the formulation of a forward model. The individuals with cerebellar dysfunction were able to accomplish the tasks and reach the target besides the increased time to complete the task and more need of corrections, decreasing the movement smoothness. This increased time and decrease in the movement smoothness seems to be related to the impairments for the planning of the movement. The individuals with cerebellar damage were not able to plan the movement with efficiency and showed longer latency and higher initial direction errors. In attempt to organize the error in the planning, to assure the accuracy and precision of movement, these individuals had to make more corrections; for that purpose, the time to accomplish the movement was increased.

The results of this study also showed that the movement direction influences the speed and the required corrections to produce an accurate movement. For the ipsilateral direction, the movement was performed faster, with higher peak velocity and less corrections than movements to the contralateral direction. This behavior was similar in individuals with cerebellar damage. The joints of the upper limb perform different functions related to the direction in which the movement is performed. Contralateral movements that cross the body midline require a greater range of motions in the joints of the shoulder and elbow, thus requiring greater interarticular coordination. Miall and King [23] describe that for targets located at about 45° in the ipsilateral direction, the elbow joint generates the main torque while the shoulder, in a subordinate way, ensures the necessary adjustments so that the target is reached. On the other hand, in the contralateral direction, the lead of the torque generation in the movement seems to reverse. This interarticular coordination seems to depend on adequate cerebellar activity [1, 12].

The upper limbs have a key role in carrying out daily activities that depend on reaching, holding, and manipulating. The coordinated interaction between the arm segments leads to the proper positioning of the hand in the space according to the specific demands of the task. Therefore, the control of the central nervous system and especially the cerebellum is necessary. This study showed that cerebellar disease leads to a loss in the planning (forward model) and execution (adaptations) of aiming movements. The planning of the movement and the formulation of a forward model are primarily affected during aiming movements. Deficits in the execution of the ongoing movements seem to be a consequence of alterations in the forward models. All findings are compatible with the role of this structure in the coordination of voluntary movement.

There were however several drawbacks of the study. The small number of participants and the heterogeneity of the sample related to the etiology of the cerebellar ataxia (degenerative and vascular etiologies) may have influenced the present results and limited its generalization. Further studies on a larger number of participants should be performed in order to properly investigate the impact of cerebellar dysfunction on upper limb functional capacity and motor performance.

## 5. Conclusion

Cerebellar disorder leads to impaired aiming movement performance related to the planning (forward model) and execution (adaptations) of movements. Such disruptions seem to be connected, primarily, to an altered movement planning and formulation of an internal model, showed by the increased reaction time and initial direction error. Impaired movement execution (adaptations) seems to be a consequence of disruptions in the formulation of an anticipatory model, leading to more adaptations which make the movement slower and with more corrections. These findings are compatible with the roles of this structure on the control of voluntary movement.

## Figures and Tables

**Table 1:** Average and frequency of sociodemographic data and physical-functional tests, related to the cerebellar (*n* = 6) and healthy (*n* = 6) groups.

Variable	Cerebellar	Healthy
Age (years)	56 (15.2)	57 (15.0)
Weight (kg)	70.5 (13.4)	79.0 (11.1)
Height (m)	1.67 (0.1)	1.71 (0.1)
IMC (kg/m^2^)	25.1 (4.0)	27.0 (5.5)
Gender^∗^		
Female	33.3 (2)	33.3 (2)
Most affected limb^∗^		
Upper right limb	66.7 (4)	—
Upper left limb	33.3 (2)	—
Etiology^∗^		
Cerebrovascular accident	50.0 (3)	—
Degenerative diseases	50.0 (3)	—
Physical-functional tests		
Palmar gripping force (kgf)	26.6 (7.2)	35.2 (9.1)
Clamp force (kgf)	3.3 (2.8)	3.2 (0.7)
Purdue Pegboard Test (hits)	5.8 (2.7)	13.4 (1.0)^∗∗^
Fugl-Meyer (score)		
Total upper limb	120.5 (4.09)	—
Specific coordination	2.83 (1.17)	—

^∗^Frequency data presented in % (*n*) but other data are presented as average (SD); ^∗∗^difference between groups: *p* = 0.0004.
